# Draft genome sequence of *Acinetobacter baumannii* strain NCTC 13423, a multidrug-resistant clinical isolate

**DOI:** 10.1186/s40793-016-0181-7

**Published:** 2016-09-01

**Authors:** Joran E. Michiels, Bram Van den Bergh, Maarten Fauvart, Jan Michiels

**Affiliations:** 1 Centre of Microbial and Plant Genetics, KU Leuven, B-3001 Leuven, Belgium; 2 Smart Systems and Emerging Technologies Unit, Department of Life Science Technologies, imec, B-3001 Leuven, Belgium

**Keywords:** Draft genome, *Acinetobacter baumannii*, Nosocomial pathogen, Multidrug resistance, Human isolate

## Abstract

*Acinetobacter baumannii* is a pathogen that is becoming increasingly important and causes serious hospital-acquired infections. We sequenced the genome of *A. baumannii* NCTC 13423, a multidrug-resistant strain belonging to the international clone II group, isolated from a human infection in the United Kingdom in 2003. The 3,937,944 bp draft genome has a GC-content of 39.0 % and a total of 3672 predicted protein-coding sequences. The availability of genome sequences of multidrug-resistant *A. baumannii* isolates will fuel comparative genomic studies to help understand the worrying spread of multidrug resistance in this pathogen.

## Introduction

*Acinetobacter baumannii* recently emerged as an increasingly important pathogen causing healthcare-associated bloodstream, urinary tract, pulmonary, and device-related infections [1]. *A. baumannii* strains are often resistant against multiple antibiotics, owing to their high intrinsic resistance and a variety of acquired resistance mechanisms [2]. Carbapenem is usually an effective treatment choice, but carbapenem-resistant strains are globally on the rise, and alternative treatment options are limited [3].

Here, we present the draft genome sequence of *A. baumannii*NCTC 13423, a strain belonging to international clone lineage II isolated from a patient in a UK hospital in December 2003 [4]. NCTC 13423 shows resistance to ampicillin, amoxicillin-clavulanic acid, aztreonam, cefepime, cefotaxime, ceftazidime, cefoxitin, piperacillin, piperacillin-tazobactam, ciprofloxacin, gentamicin, and sulbactam [4]. Although originally reported as carbapenem-sensitive, a later report classified it to be also carbapenem-resistant [5]. Additionally, this strain is highly virulent and a strong biofilm producer [6].

## Organism information

### Classification and features

**Fig. 1:**
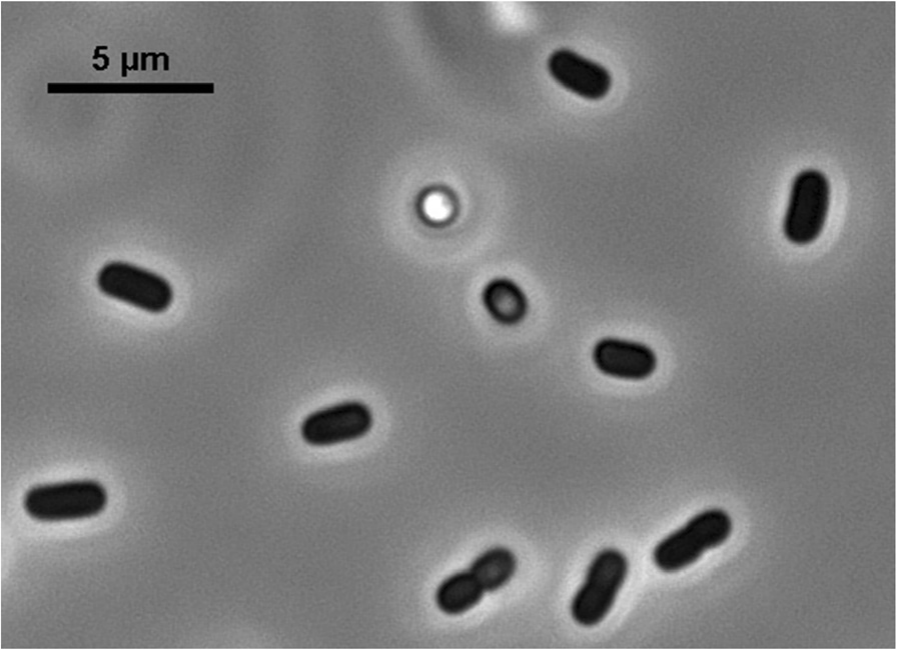
Phase-contrast micrograph of *A. baumannii* NCTC 13423

**Fig. 2:**
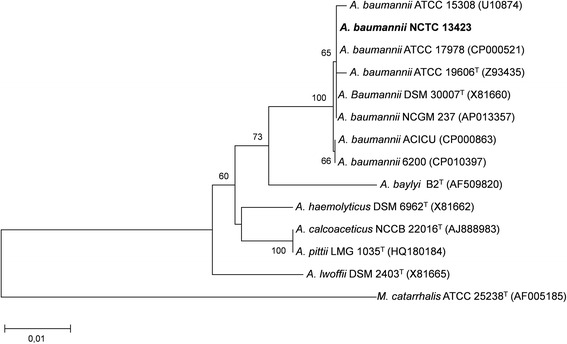
16S rRNA phylogenetic analysis showing the evolutionary relationship between *A. baumannii* NCTC 13423 and related type (^T^) and non-type *A. baumannii* strains and *Acinetobacter* species. *Moraxella catarrhalis* was used as an outgroup. Genbank accession numbers of the aligned sequences are indicated between brackets. Sequence alignment was performed using MUSCLE [27], and a neighbour-joining algorithm using the Kimura 2-parameter distance model was used to construct a phylogenetic tree in MEGA (version 7) [28]. The rate variation among sites was modelled with a gamma distribution (shape parameter = 1). The optimal tree with the sum of branch lengths = 0.1583 is shown, and bootstrap support values above 60 % (1000 replicates) are indicated next to the branches

Bacteria in the genus *Acinetobacter* are Gram-negative, strictly aerobic, nonfermenting, nonmotile, catalase-positive, oxidase-negative coccobacilli [7] (Table 1). The genus *Acinetobacter* has gone through many taxonomic changes over the years, and the species *A. baumannii* has only been officially recognized since 1986 [8, 9]. *A. baumannii* belongs to the family *Moraxellaceae*, order *Pseudomonadales*, class *Gammaproteobacteria*, and phylum *Proteobacteria*. *Acinetobacter* species are ubiquitous organisms, widely distributed in nature, and can be recovered from virtually any soil or water sample. However, *A. baumannii* seems to be an exception to this rule, as it currently has no known habitats except the hospital [10]. Microscopically, they are often observed as pairs of cells (Fig. 1). *A. baumannii* can withstand prolonged desiccation, allowing it to survive on dry surfaces and probably contributing to its persistent residence in hospital settings [11]. A phylogenetic tree based on 16S rDNA sequences showed strong clustering with other *A. baumannii* strains (Fig. 2).

**Table 1:** Classification and general features of *Acinetobacter baumannii* strain NCTC 13423 according to the MIGS recommendations [12]

MIGS ID	Property	Term	Evidence code^a^
	Classification	Domain *Bacteria*	TAS [29]
		Phylum *Proteobacteria*	TAS [30]
		Class *Gammaproteobacteria*	TAS [31, 32]
		Order *Pseudomonadales*	TAS [33, 34]
		Family *Moraxellaceae*	TAS [35]
		Genus *Acinetobacter*	TAS [34, 36]
		Species *Acinetobacter baumannii*	TAS [8]
		Strain NCTC 13423	NAS
	Gram stain	Negative	TAS [8]
	Cell shape	Coccobacillus	TAS [8]
	Motility	Non-motile	TAS [37]
	Sporulation	Non-sporulating	TAS [8]
	Temperature range	Mesophilic	TAS [38]
	Optimum temperature	37 °C	TAS [38]
	pH range; Optimum	Unknown	NAS
	Carbon source	Chemoorganoheterotrophic; citrate, lactate, ethanol, glutarate, malate, aspartate, tyrosine, 2,3-butanediol, 4-aminobutyrate	TAS [8]
MIGS-6	Habitat	Hospital	NAS
MIGS-6.3	Salinity	Unknown	NAS
MIGS-22	Oxygen requirement	Strictly aerobic	TAS [8]
MIGS-15	Biotic relationship	Free-living	TAS [8]
MIGS-14	Pathogenicity	Pathogenic	TAS [4]
MIGS-4	Geographic location	United Kingdom	TAS [4]
MIGS-5	Sample collection	12/2003	TAS [4]
MIGS-4.1	Latitude	Unknown	NAS
MIGS-4.2	Longitude	Unknown	NAS
MIGS-4.4	Altitude	Unknown	NAS

^a^Evidence codes, *IDA* inferred from direct assay, *TAS* traceable author statement (i.e., a direct report exists in the literature), *NAS* non-traceable author statement (i.e., not directly observed for the living, isolated sample, but based on a generally accepted property for the species, or anecdotal evidence). These evidence codes are from the Gene Ontology project [39]

## Genome sequencing information

### Genome project history

The strain NCTC 13423 was isolated in 2003 in the United Kingdom from a repatriated casualty of the Iraq conflict [4], and was selected for sequencing because of its multidrug-resistant and virulence characteristics. Sequencing was carried out at the EMBL GeneCore facility (Heidelberg, Germany). Sequences were assembled using CLC Genomics Workbench (version 7.5.1) and annotated using NCBI’s Prokaryotic Genome Annotation Pipeline (PGAP). This draft whole-genome sequence has been deposited at DDBJ/ENA/GenBank under the accession LOHD00000000. The project information, and its association with MIGS version 2.0 [12], is summarised in Table 2.

**Table 2:** Project information

MIGS ID	Property	Term
MIGS-31	Finishing quality	High-quality draft
MIGS-28	Libraries used	One paired-end Illumina library (Nextera)
MIGS-29	Sequencing platforms	Illumina HiSeq 2000
MIGS-31.2	Fold coverage	203
MIGS-30	Assemblers	CLC NGS Cell 7.5.1
MIGS-32	Gene calling method	GeneMarkS+
	Locus Tag	AUC58
	Genbank ID	LOHD00000000
	GenBank Date of Release	2016/02/26
	GOLD ID	-
	BIOPROJECT	PRJNA305394
MIGS-13	Source Material Identifier	NCTC 13423
	Project relevance	Medical

### Growth conditions and genomic DNA preparation

Cultures for DNA isolation were inoculated from a single colony on LB agar in 5 ml lysogeny broth and grown overnight at 37 °C with orbital shaking (200 rpm). DNA was isolated using the DNeasy Blood&Tissue Kit (Qiagen) following the manufacturer’s instructions and pre-treatment protocol for Gram-negative bacteria. DNA concentration and purity were assessed using the Nanodrop ND-1000 spectrophotometer and Qubit fluorometer (ThermoFisher Scientific).

### Genome sequencing and assembly

Sequencing was performed using the Nextera DNA Library Preparation Kit with the Illumina HiSeq 2000 platform (100 bp, paired-end) at the EMBL GeneCore facility (Heidelberg, Germany). The read library contained a total of 8,765,016 sequences in pairs. Sequence data was analysed using Qiagen’s CLC Genomics Workbench (version 7.5.1). First, reads were trimmed for quality (score limit 0.05) and ambiguous nucleotides (maximum 2 ambiguities). Next, *de novo* assembly was performed (mismatch cost: 2, deletion cost: 3, insertion cost: 3, length fraction: 0.5, similarity fraction: 0.8), yielding 196 contigs (minimum length 200 bp) with an average coverage of 203x. Contigs averaged 20,092 bp in length (N50 of 111,328 bp). The total length of the draft genome is 3,937,944 bp with a GC-content of 39.0 %.

### Genome annotation

All contigs were annotated using NCBI’s Prokaryotic Genome Annotation Pipeline (PGAP). The Batch Web CD-Search Tool from NCBI [13] was used to identify Pfam domains [14] in the predicted protein sequences. Classification of predicted proteins in Clusters of Orthologous Groups (COG) functional categories [15] was done with the WebMGA web server for metagenomic analysis [16]. Signal peptides, transmembrane domains, and CRISPR repeats were predicted using the SignalP 4.1 server [17], the TMHMM server [18], and the CRISPRFinder tool [19], respectively. Only confirmed and not questionable CRISPR hits were taken into account.

## Genome properties

Table 3 summarises the properties of the draft genome. Reads were assembled into 196 contigs, totalling 3,937,944 bp with a 39.0 % GC-content. PGAP predicted a total number of 3875 genes, including 3672 protein coding genes (totalling 3,384,768 base pairs), 135 pseudo genes, and 68 RNA genes (64 tRNA, 3 rRNA, and 1 ncRNA). 75.17 % of the protein-coding genes had a putative function assigned, the remainder was annotated as a hypothetical protein. Additional characteristics of the predicted genes are given in Table 3, and Table 4 shows their distribution amongst the different functional COG categories.

**Table 3:** Genome statistics

Attribute	Value	% of Total
Genome size (bp)	3,937,944	100
DNA coding (bp)	3,384,768	85.95
DNA G + C (bp)	1,537,664	39.05
DNA scaffolds	196	100
Total genes	3875	100
Protein coding genes	3672	94.76
RNA genes	68	1.75
Pseudo genes	135	3.48
Genes in internal clusters	-	-
Genes with function prediction	2913	75.17
Genes assigned to COGs	3174	81.91
Genes with Pfam domains	3,002	77.47
Genes with signal peptides	313	8.08
Genes with transmembrane helices	882	22.76
CRISPR repeats	0	-

**Table 4:** Number of genes associated with general COG functional categories

Code	Value	%age	Description
J	177	4.82	Translation, ribosomal structure and biogenesis
A	1	0.03	RNA processing and modification
K	272	7.41	Transcription
L	125	3.40	Replication, recombination and repair
B	0	0.00	Chromatin structure and dynamics
D	32	0.87	Cell cycle control, Cell division, chromosome partitioning
V	40	1.09	Defense mechanisms
T	97	2.64	Signal transduction mechanisms
M	193	5.26	Cell wall/membrane biogenesis
N	42	1.14	Cell motility
U	88	2.40	Intracellular trafficking and secretion
O	112	3.05	Posttranslational modification, protein turnover, chaperones
C	202	5.50	Energy production and conversion
G	138	3.76	Carbohydrate transport and metabolism
E	288	7.84	Amino acid transport and metabolism
F	81	2.21	Nucleotide transport and metabolism
H	131	3.57	Coenzyme transport and metabolism
I	182	4.96	Lipid transport and metabolism
P	185	5.04	Inorganic ion transport and metabolism
Q	97	2.64	Secondary metabolites biosynthesis, transport and catabolism
R	406	11.06	General function prediction only
S	285	7.76	Function unknown
-	498	13.56	Not in COGs

The total is based on the total number of protein coding genes in the genome

## Insights from the genome sequence

Functional analysis of the genome sequence by RAST annotation [20] revealed *A. baumannii* ACICU as the closest related sequenced neighbor. *A. baumannii* ACICU is an epidemic, multidrug-resistant strain isolated from a hospital outbreak in Rome [21]. The high genetic relatedness between *A. baumannii* ACICU and *A. baumannii*NCTC 13423 was confirmed by calculating their two-way average amino acid identity (AAI), which was 99.30 % based on 3360 protein sequences [22]. Indicative for the multidrug-resistant phenotype, annotations by RAST included six different β-lactamase enzymes, among which two AmpC-type β-lactamases (class C), a metallo-β-lactamase (class B), two class A β-lactamases (of which one TEM-type broad-spectrum β-lactamase) and an oxa-51 like carbapenemase (class D). Using TAfinder, a web-based tool to identify type II toxin-antitoxin (TA) loci in bacterial genomes [23], we predicted the presence of 12 type II TA modules in the *A. baumannii*NCTC 13423 draft genome. Considering only TAfinder hits with normalized homology scores (H-value) > 0.5, five putative TA modules remain, three of which are also present in the genome of *A. baumannii* ACICU. Interestingly, *A. baumannii* has been reported to form antibiotic-tolerant persister cells [24, 25], and these TA modules might play a role in their formation [26].

## Conclusions

We determined the draft genome sequence of the highly virulent, multidrug-resistant *A. baumannii*NCTC 13423 clinical isolate. The availability of genomic sequences of clinical *A. baumannii* isolates from a variety of locations and sources will benefit comparative genomic studies to better understand the worrying spread of multidrug resistance in this pathogen.

## Abbreviations

COG: Clusters of orthologous groups
PGAP: Prokaryotic genome annotation pipeline
